# Role of miR-199a-5p in the post-transcriptional regulation of ABCA1 in response to hypoxia in peritoneal macrophages

**DOI:** 10.3389/fcvm.2022.994080

**Published:** 2022-11-03

**Authors:** Juan Francisco Aranda, Ana Pérez-García, Marta Torrecilla-Parra, Mario Fernández-de Frutos, Yolanda Martín-Martín, Pedro A. Mateos-Gómez, Virginia Pardo-Marqués, Rebeca Busto, Cristina M. Ramírez

**Affiliations:** ^1^Department of Basic Medical Sciences, CEU San Pablo University, CEU Universities, Madrid, Spain; ^2^IMDEA Research Institute of Food and Health Sciences, Madrid, Spain; ^3^Department of Systems Biology, School of Medicine and Health Sciences, University of Alcalá, Madrid, Spain; ^4^Department of Clinical Biochemistry, Hospital Universitario Ramón y Cajal, IRYCIS, Madrid, Spain

**Keywords:** miRNAs, hypoxia, ABCA1, atherosclerosis, macrophage, cholesterol efflux

## Abstract

Hypoxia is a crucial factor contributing to maintenance of atherosclerotic lesions. The ability of ABCA1 to stimulate the efflux of cholesterol from cells in the periphery, particularly foam cells in atherosclerotic plaques, is an important anti-atherosclerotic mechanism. The posttranscriptional regulation by miRNAs represents a key regulatory mechanism of a number of signaling pathways involved in atherosclerosis. Previously, miR-199a-5p has been shown to be implicated in the endocytic and retrograde intracellular transport. Although the regulation of miR-199a-5p and ABCA1 by hypoxia has been already reported independently, the role of miR-199a-5p in macrophages and its possible role in atherogenic processes such us regulation of lipid homeostasis through ABCA1 has not been yet investigated. Here, we demonstrate that both ABCA1 and miR-199a-5p show an inverse regulation by hypoxia and Ac-LDL in primary macrophages. Moreover, we demonstrated that miR-199a-5p regulates ABCA1 mRNA and protein levels by directly binding to its 3’UTR. As a result, manipulation of cellular miR-199a-5p levels alters ABCA1 expression and cholesterol efflux in primary mouse macrophages. Taken together, these results indicate that the correlation between ABCA1-miR-199a-5p could be exploited to control macrophage cholesterol efflux during the onset of atherosclerosis, where cholesterol alterations and hypoxia play a pathogenic role.

## Introduction

Atherosclerosis is the major cause of cardiovascular disease, which is the leading cause of death worldwide (1). It is characterized by the progressive accumulation of lipids, inflammatory cells and fibrous material in the sub-endothelial space of large arteries (2). Macrophages play a central role in the development of this disease, since these cells are the key mediators of the establishment, progression and, ultimately, instability of atherosclerotic plaques (3). Among the plethora of mechanisms involved in the development and progression of atherosclerosis, hypoxia has been described as a relevant factor for the maintenance of atherosclerotic lesions. Indeed, in human atherosclerotic plaques, the hypoxic regions are enriched in macrophages and foam cells, key pathological hallmarks of the plaques (4, 5) and it has been recognized as a trigger of inflammation (6). The transcription factor Hypoxia Inducible Factor-1 (HIF-1), which is crucial in the cellular adaptation to hypoxia (7), colocalizes with macrophages and it is directly associated with inflammatory plaque phenotype, angiogenesis, and hemorrhage in humans (5, 8, 9). Furthermore, HIF-1 is also known to regulate key factors relevant to atherosclerosis, such as genes involved in apoptosis, nitric oxide pathway, inflammation, and intracellular redox homeostasis and lipid metabolism, including ATP binding cassette subfamily A member 1 (ABCA1) (10–13). In addition, it has been identified a potential binding site for HIF-1 in the ABCA1 promoter and that its expression and cholesterol efflux are regulated by HIF-1 under hypoxic conditions (14, 15). Interestingly, other study shows that Liver X Receptor (LXR), the main transcription factor regulating ABCA1, is also increased by hypoxia (16). Besides to transcriptional control of hypoxia, novel alternative mechanisms mediated by miRNAs have been described in the regulation of this process under different contexts related with human diseases, such as cancer or metabolic disorders (17, 18).

MiRNAs (miRs) are a class of small non-coding RNAs made up of 18–25 nucleotides in length that regulate gene expression at the posttranscriptional level and play important roles in many fundamental biological functions such as cell growth, differentiation, development, and apoptosis (19). This regulatory control is carried out by base pairing with complementary regions, mainly within the 3’-untranslated regions (3′UTRs) of target mRNAs, thus promoting mRNA degradation, translational repression, or both (19–21). In the last 10 years, a vast number of studies from different laboratories, including our own, have demonstrated a critical role of miRNAs in the regulation of many metabolic diseases by interfering with key genes involved in diabetes, Alzheimer’s disease or atherosclerosis (17, 22, 23). In this context, miRNAs that regulate ABCA1 have been a great focus of attention, given the role of this protein in the regulation of cholesterol and lipoprotein metabolism, which underlies the above-mentioned diseases. Some of these miRNAs include miR-33 (24, 25), miR-758 (26), miR-144 (27), miR-27b (28), miR-148a (29), and miR-106b (30). It is well stablished that hypoxia’s effects on lipid metabolism are mediated in part by HIF-1. Interestingly, ABCA1 has been described as one of the strongest upregulated genes in primary human vascular endothelial cells and in human and mouse macrophages in response to hypoxia (4, 14, 31). However, to date, there are few evidences of the posttranscriptional regulation of ABCA1 mediated by hypoxia (32, 33). Here, we show that miR-199a-5p expression levels are inhibited during hypoxia or hypoxia-mimicking conditions compared to normoxia, and that it can directly bind to the 3’UTR of ABCA1 inhibiting its expression and function by blunting cholesterol efflux to ApoAI.

The miR-199a/b family is composed of three members: miR-199a1, miR-199a2, and miR-199b, located within the Dynamin-2 (DNM2), DNM3 and DNM1 genes, respectively (34). MiR-199a/b mature sequences are highly conserved across species and share the same seed sequence, thereby potentially targeting the same group of genes. We have previously reported that miR-199a-5p regulates endocytic transport by controlling important mediators such as Low Density Lipoprotein Receptor (LDLR) and Caveolin-1 (Cav-1) (34) and retrograde transport as well as autophagosome formation and lysosomal function (35). miR-199a-5p has been also shown to participate in the regulatory mechanism of hypoxia by targeting HIF-1, and to influence cellular proliferation survival and cell death (36, 37). However, little is known about the association between miR-199a-5p and hypoxia in the context of cholesterol metabolism, specifically in macrophages. In this report, we have identified the hypoxamiR miR-199a-5p, as a novel regulator of cholesterol efflux through posttranscriptional regulation of ABCA1 in primary mouse macrophages and cholesterol-laden-macrophages that resemble foam cells present in the atherosclerotic plaques.

## Materials and methods

### Cell culture and treatments

Peritoneal macrophages from adult male WT (3 months old) mice were harvested by peritoneal lavage 4 days after intraperitoneal injection of thioglycolate (3% w/v). Cells were plated in RPMI 1,640 medium supplemented with 10% fetal bovine serum, 100 U/ml penicillin, and 100 U/ml streptomycin. After 4 h, non-adherent cells were washed out, and macrophages were incubated in fresh medium containing DMEM, 20% fetal bovine serum, and 20% L-cell conditioned medium overnight, and cells were maintained in culture as an adherent monolayer adding fresh medium every day. For hypoxia experiments, peritoneal macrophages were placed in a hypoxia chamber (Biospherix, Lacona, NY) (0.5% oxygen, 5% CO_2_) at 37^°^C. Control normoxic cells were kept in a standard cell culture incubator (37^°^C, 5% CO_2_). Depending on the experiment, hypoxia conditions were maintained for 6, 12, 24, or 48 h. After these time points, cells were collected and immediately processed for total RNA or protein isolation as indicated before (22). For some experiments, macrophages were treated with LXR ligand T0901317 (T090) 3 μM for 12 h and Acetylated LDL (Ac-LDL) (120 μg/mL) for 24 h. CoCl_2_ were used at 100 μM to mimic hypoxia conditions in cultured primary macrophages.

### Bioinformatic analysis miRNA-199 target genes

Predicted target genes for miR-199a-5p were identified using TargetScan 6.2. GeneMania software^1^ and Gtex portal^2^ were used to represent networks of predicted targets involved in Atherosclerosis and tissue gene expression for MIR199.

### Transfection of miRNA mimics, miRNA inhibitors and target site blockers

Cells (∼70% confluence) were transfected with 40 nM miRIDIAN miRNA mimic (miR-199a-5p), or with 60 nM miRIDIAN miRNA inhibitor (Inh-miR-199a-5p) (Dharmacon) utilizing Lipofectamine *™* RNAimax (Invitrogen) and studied 48 h later. In all experiments, an equal concentration of a non-targeting control mimic sequence (CM) or inhibitor-negative control (CI) sequence was used as a control for non-sequence specific effects in miRNA experiments. Verification of miR-199a-5p overexpression and specific knockdown was determined using qPCR as described below. For some experiments 40 nM of scramble target site blocker (Ctrl TSB) or miR-199-5p TSB (miRCURY LNA miRNA Power Target Site Blockers, Quiagen) were transfected with 20 nM of CM or miR-199a-5p mimics for 48 h.

### Ribonucleic acid isolation and quantitative real-time polymerase chain reaction

Total RNA from primary macrophages were isolated using TRIzol reagent (Invitrogen) according to the manufacturer’s protocol. For mRNA quantification, 1 μg of total RNA was reverse transcribed to cDNA using iScript RT Supermix (Bio-Rad), following the manufacturer’s protocol. Quantitative real-time PCR was performed in triplicate using iQ SYBR green Supermix (BioRad) on Real-Time Detection System (Eppendorf). The mRNA level was normalized to 18S as a housekeeping gene. Primer sequences are available upon request. For miRNA quantification, total RNA was reverse transcribed using the miScript II RT Kit (Qiagen). Specific primers for mature miR-199a-5p, miR-199b-5p, miR-33a-5p, miR-758-5p, miR-144-5p, and miR-210-5p (Qiagen) were used and values were normalized to SNORD68 (Qiagen) as a housekeeping gene.

### Western blot analysis

Western blot analysis was assessed as previously shown (22). Very briefly, cells were lysed in ice-cold buffer containing 50 mM Tris-HCl, pH 7.4, 0.1 mM EDTA, 0.1 mM EGTA, 1% NP-40, 0.1% sodium deoxycholate, 0.1% SDS, 100 mM NaCl, 10 mM NaF, 1 mM sodium pyrophosphate, 1 mM sodium orthovanadate, 1 mM Pefabloc, and 2 mg/ml protease inhibitor cocktail (Roche Diagnostics Corp.). Protein concentrations were determined using the DC Protein assay kit (Bio-Rad Laboratories). Cell lysates containing 12.5–25 μg of protein were analyzed by SDS-PAGE and immunoblotting. Primary antibodies used include the following: ABCA1 (#ab18180, Abcam), ABCG1 (#NB400-132), Cav-1 (#SC-894) and HSP90 (#610419, BD Bioscience). Secondary antibodies were fluorescence-labeled antibodies and bands were visualized using the Odyssey Infrared Imaging System (LI-COR Biotechnology). Densitometry analysis of the Western blots was carried out by using ImageJ software from the NIH.

### 3′TR luciferase reporter assays

The cDNA fragments corresponding to the entire 3′UTR of human ABCA1 mRNAs were amplified by RT-PCR using the following primers: 5′-AGCGGCCGCTTTCTGTAGACCAAC AGAACTGTCA-3′ (*Not*I) and 5′- AACTCGAGAGAATCCTG TTCATACGGGG-3′ (*Xho*I) from total RNA extracted from cells (26). The PCR product was directionally cloned downstream of the RLuc open reading frame of the psiCHECK2 TM vector (Promega) that also contains a constitutively expressed firefly luciferase gene, which is used to normalize transfections. Site-directed mutations in the seed region of predicted miR-199a-5p sites within the 3′UTRs were generated using Quickchange Multi site directed mutagenesis kit (Agilent) according to the manufacturer’s protocol. All constructs were confirmed by sequencing. COS-7 cells were plated into 12-well plates and cotransfected with 1 μg of the indicated 3′UTR luciferase reporter vectors and the miR-199a-5p mimic or negative control mimic (CM) (Dharmacon) using Lipofectamine 2000 (Invitrogen). For some experiments, 40 nM of Ctrl TSB or miR-199a-5p TSB were transfected with 20 nM of CM or miR-199a-5p mimics for 24 h. Luciferase activity was measured using the Dual-Glo Luciferase Assay System (Promega). Renilla luciferase activity was normalized to the corresponding firefly luciferase activity and plotted as a percentage of the control (CM). Experiments were performed in triplicate wells of 12-well plates and repeated at least three times.

### Cholesterol efflux assays

Mouse peritoneal macrophages (1 × 10^6^/well) were transfected with either 40 nM miRIDIAN miRNA mimic (miR-199a-5p), with 60 nM miRIDIAN miRNA inhibitor (Inh-miR-199a-5p) or control mimic sequence (CM) 48 h prior to loading with 0.5 μCi/ml ^3^H-cholesterol for 24 h with or without T090 (3 μM) for 12 h. Then, cells were washed twice with PBS and incubated in RPMI supplemented with 2 mg/ml fatty-acid free BSA (FAFA media) in the presence of 2 μM of the Acetyl-Coenzyme A Acetyltransferase (ACAT) inhibitor Sandoz (Sigma Aldrich) for 4 h prior to the addition of 50 μg/ml of human ApoA1 in FAFA media. Supernatants were collected after 6 h, and radioactivity content was expressed as a percentage of ^3^H-cholesterol in the media/total cell ^3^H-cholesterol content (total effluxed ^3^H-cholesterol + cell-associated ^3^H-cholesterol) (27). Experiments were repeated at least 3 times. Cholesterol efflux experiments were performed in normoxic conditions.

### Fluorescence microscopy

For Bodipy 493 staining, macrophages were plated into culture dishes containing coverslips and after 24 h of treatment with Ac-LDL (120 μg/mL), the cell were washed twice in PBS and incubated with 2 μM Bodipy 493 staining solution in PBS for 15 min at 37^°^C. Then, cells were washed again in PBS twice and fixed in 4% PFA for 30 min at room temperature. After washing cells were mounted Vectashield mounting media with Dapi and images were capture using an epifluorescence microscope (Carl Zeiss scanning microscope Axiovert 200M imaging system).

### Statistical analysis

*In vitro* experiments were routinely repeated at least three times unless otherwise noted. Data are expressed as mean ± SEM unless otherwise indicated. Statistical differences were measured using an unpaired two-sided Student’s *t*-test, one-way ANOVA with Bonferroni correction for multiple comparisons or log-rank test when appropriate. Normality was checked using the Kolmogorov-Smirnov test. A non-parametric test (Mann-Whitney) was used when data did not pass the normality test. A value of *p* ≤ 0.05 was considered statistically significant. Data analysis was performed using GraphPad Prism Software Version 7 (GraphPad, San Diego, CA).

## Results

### MiR-199a-5p targets key elements involved in atherosclerosis

**FIGURE 1:**
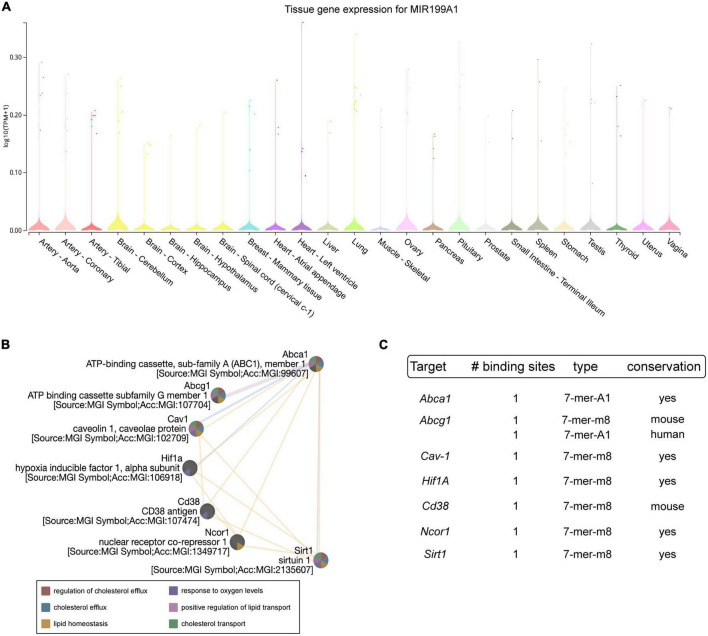
Analysis of miR-199a-5p targets involved in Atherosclerosis. **(A)** Representation of miR-199a-5p expression in human tissue using Gtex Portal. Expression values are shown in TPM (Transcripts Per Million), calculated from a gene model with isoforms collapsed to a single gene. No other normalization steps have been applied. Box plots are shown as median and 25th and 75th percentiles and points are displayed as outliers if they are above or below 1.5 times the interquartile range. **(B)** Bioinformatic analysis of prominent miR-199a-5p target genes obtained thought TargetScan 6.2. involved in atherosclerosis using GeneMania. Blue lines indicate pathway co-localization, yellow lines indicate predicted and purple lines indicate co-expression. **(C)** Table of human predicted targets genes of miR-199a-5p involved in atherosclerosis obtained using TargetScan 6.2., showing the number and type of the putative binding sites and the conservation in mouse and human.

MiR-199 is a highly conserved family of miRNAs consisting of three members, miR-199-a1, miR-199-a2 and miR-199-b, that are encoded within DNM genes (34). The analysis obtained with Gtex portal (see text footnote 2) of miR-199a-5p expression in different human tissues shows its abundancy in the aorta and in the heart (Figure 1A). To determine which molecular pathways would be affected by miR-199a-5p, we performed predicted target genes analysis using bioinformatic tools for miRNA target predictions^3^. Among the different processes in which miR-199a-5p participate, several targets are implicated in atherosclerosis including ABCA1, ABCG1, Cav-1, HIF1A, CD38, NCOR1, and SIRT1 in human and mouse (Figure 1B). The number, type and conservation of predicted sites for these target genes are shown in Figure 1C. These initial data led us to explore the potential correlation between miR-199a-5p with one of the most widely studied genes in the context of cardiovascular disease and cholesterol metabolism, ABCA1.

### MiR-199a-5p is downregulated during hypoxia and other atherogenic stimuli in primary mouse macrophages

**FIGURE 2:**
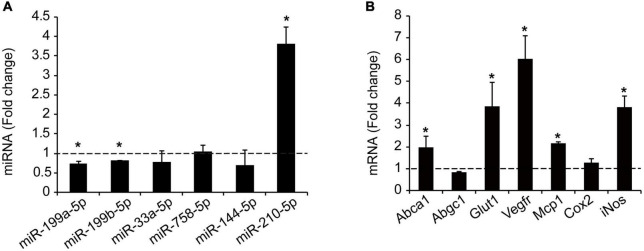
Hypoxia regulates miRNAs and cholesterol transporter ABCA1. **(A)** qRT-PCR analysis of miR-199a-5pa, miR-199b-5p, miR-33a-5p, miR-758-5p, and miR-144-5p in peritoneal macrophages exposed to hypoxic conditions. HypoxamiR miR-210-5p was used as a positive control. **(B)** qRT-PCR analysis of Abca1, Abcg1, Glut-1, Vegfr, Mcp1, Cox-2, and iNos expression in mouse peritoneal macrophages during hypoxia conditions. Data are expressed as relative expression levels and correspond to the means ± SEM from three independent experiments performed in triplicate. *P < 0.05, significantly different from control normoxic cells.

**FIGURE 3:**
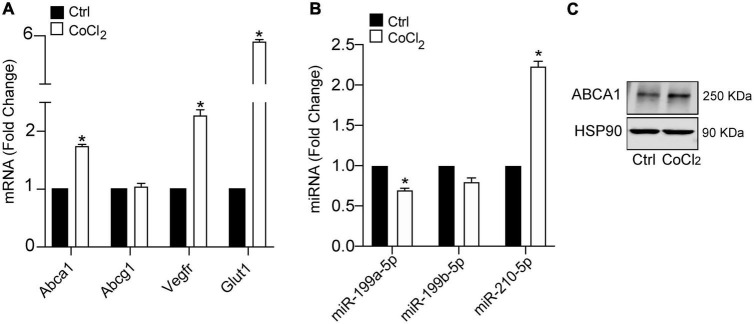
Hypoxia mimicking condition dowregulates miR-199a-5p expression. **(A)** qRT-PCR analysis of miR-199a-5p, miR-199b-5p, and miR-210-5p mouse peritoneal macrophages stimulated with CoCl_2_ (100 μM) that mimics hypoxia conditions. **(B)** qRT-PCR analysis Abca1, Hifα, Vegfr, and Glut-1 expression levels in was used as a positive control for hypoxia. **(C)** Representative Western Blot analysis of ABCA1 in mouse peritoneal macrophages stimulated with CoCl_2_ (100 μM) for 24 h. HSP90 was used as a loading control. Data are expressed as relative expression levels and correspond to the means ± SEM from three independent experiments performed in triplicate. *P < 0.05, significantly different from control (Ctrl) non-treated cells.

**FIGURE 4:**
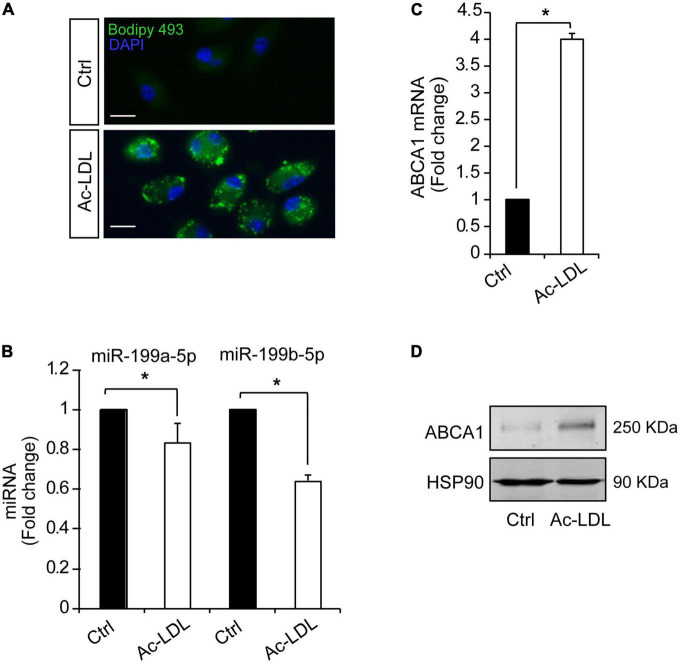
Cholesterol loading in peritoneal macrophages downregulates miR-199a-5p and upregulates ABCA1. **(A)** Representative images showing lipid droplets in mouse peritoneal macrophages treated with Ac-LDL for 24 h and stained with Bodipy 468 (green). Nuclei were stained with DAPI (blue). These experiment were performed 3 independent times. Scalebar: 10 μm. **(B,C)** qRT-PCR analysis of miR-199a-5p, miR-199b-5p **(B)** and Abca1 **(C)**, expression levels in mouse peritoneal macrophages stimulated with Ac-LDL (120 μg/mL) for 24 h. Data are expressed as relative expression levels and correspond to the means ± SEM from three independent experiments performed in triplicate. *P < 0.05, significantly different from control (Ctrl) cells. **(D)** Representative Western blot of ABCA1 in mouse peritoneal macrophages stimulated with Ac-LDL. HSP90 was used as a loading control.

Hypoxia plays a key mechanistical role during atherosclerosis and upregulates ABCA1 in macrophages through HIF1α transcription factor (14). We decided to investigate whether it could regulate the expression of different miRNAs that we had previously reported to regulate cholesterol metabolism, lipid trafficking and atherosclerosis. To do so, we analyzed a panel of miRNAs including miR-33a-5p, miR-758-5p, and miR-144-5p in mouse primary peritoneal macrophages under hypoxia vs. normoxia conditions. We also included miR-199a-5p as previously shown to be involved in hypoxia (14, 15). Among the miRNAs analyzed, miR-199a-5p and miR-199b-5p were significantly downregulated during hypoxia, while no significant changes were found for miR-33a-5p, miR-758-5p, or miR-144-5p (Figure 2A). Conversely, miR-210-5p, a well-known upregulated miRNA under hypoxia conditions, was included as a positive control for these analysis (38). Correlating with the downregulation of miR-199a-5p during hypoxia we observed an increase in *Glut-1*, *Vegf*, *Mcp1*, *iNos*, all of them are known to be upregulated in hypoxia in the cell as an adaptive mechanism which allow survival and use of different fuel in this stress condition. We also observed a marked increase of *Abca1* mRNA levels (Figure 2B). Likewise, macrophages treated with CoCl_2_ showed similar results (Figure 3). We tested the two best responsive genes previously assessed in mouse peritoneal macrophages exposed to hypoxia to corroborate the efficacy of CoCl_2_. In these experiments, Vegfr and glut1 were significantly increased (Figure 3A), as well as ABCA1, while miR-199s were downregulated (Figure 3B). These effects were accompanied by ABCA1 protein increased under CoCl_2_ treatment (Figure 3C). To further explore the potential inverse correlation between ABCA1 and miR-199a-5p under atherogenic mimicking conditions *in vitro* we treated mouse peritoneal macrophages with Acetylated-LDL (Ac-LDL) for 24 h (Figure 4A). Interestingly, cholesterol-loaded macrophages showed a significantly decrease in miR-199a-5p and b-5p expression (Figure 4B), whereas as expected, Abca1 mRNA and protein levels were upregulated under these conditions (Figures 4C,D). Overall, these results indicate that both pro-atherogenic stimuli, hypoxia and cholesterol overload in peritoneal macrophages, have similar opposite effects on miR-199a-5p and ABCA1 expression, suggesting a potential regulation of ABCA1 by miR-199a-5p during hypoxia and atherosclerosis.

### MiR-199a-5p gradual downregulation inversely correlates with ABCA1 expression in mouse macrophages during hypoxia

**FIGURE 5:**
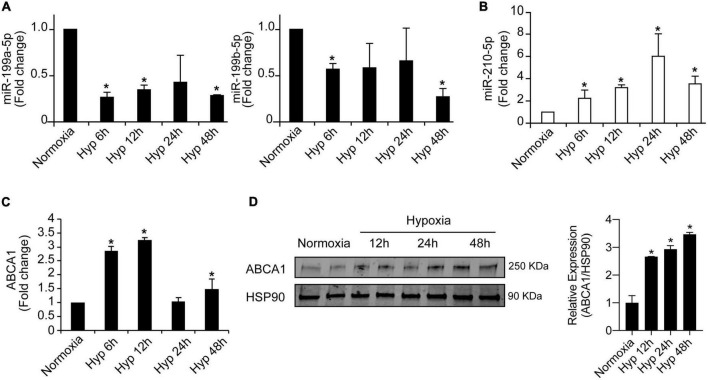
Inverse regulation of miR-199a-5p and ABCA1 during hypoxia. **(A)** qRT-PCR analysis of miR-199a-5p, and miR-199b-5p expression levels in mouse peritoneal macrophages stimulated with hypoxia at the indicated time points. **(B)** miR-210-5p was used as a positive control of hypoxia. **(C)** qRT-PCR analysis of ABCA1 in mouse peritoneal macrophages subjected to hypoxia at the indicated time points. **(D)** Representative Western blot of ABCA1 in mouse peritoneal macrophages under hypoxia at the indicated time points. HSP90 was used as a loading control. Right panel shows relative ABCA1 protein expression normalized to HSP90 (*n* = 3). Data are expressed as relative expression and correspond to the means ± SEM from three independent experiments. *P < 0.05, significantly different from cells in normoxia.

To further explore the potential regulation of miR-199 family members we analyzed the levels of miR-199a-5pa and miR-199b-5p in mouse macrophages under a time course of hypoxia. The analysis shown in Figure 5A indicates that while both miR-199 family members are significantly downregulated at 6 and 24 h, miR-199a-5p showed a more consistent and gradual downregulation correlating with the increased exposure to hypoxic conditions. MiR-210-5p was used as positive control of hypoxic experiment in peritoneal macrophages (Figure 5B). Interestingly, as opposed to miR-199a-5p behavior, ABCA1 mRNA and protein levels showed a significant increase while the hypoxia exposition was increasingly longer (Figures 5C,D). Similar results were found in bone marrow derived macrophages. Simultaneous stimulation of mouse peritoneal macrophages with hypoxia and cholesterol loading did not show any distinguishable additive effect in the conditions we assessed (Supplementary Figure 1). Given these results, we decided to explore the potential posttranscriptional regulation of ABCA1 by miR-199a-5p in mouse peritoneal macrophages.

### MiR-199a-5p regulates ABCA1 expression at the posttranscriptional level

**FIGURE 6:**
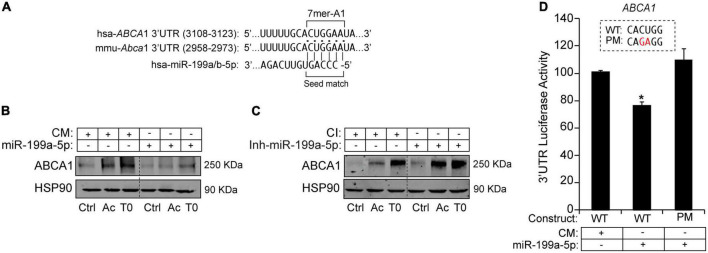
MiR-199a-5p suppresses ABCA1 expression. **(A)** Human and mouse ABCA1 3′UTRs sequences containing target sites for miR-199a/b-5p. Highlighted in bold are the sequences for miR-199a/b-5p-binding site and seed match. **(B,C)** Representative Western blot analysis of ABCA1 in mouse peritoneal macrophages transfected with CM and miR-199a-5p or Ctrl-Inh or Inh-miR-199a-5p and stimulated with T090 3 μM for 12 h and Ac-LDL for 24 h. HSP90 was used as a loading control. Dashed lines separates different transfection conditions within the same Western blot. Data are expressed as relative expression levels and correspond to the means ± SEM from three independent experiments performed in triplicate. *P < 0.05, significantly different from CM or CI without treatment. **(D)** Luciferase reporter activity in COS-7 cells transfected with CM or miR-199a-5p mimic and the ABCA1 3’UTR [wild type (WT)] or the constructs containing the indicated point mutations (PM). Nucleotides highlighted in red indicate the point mutations in the miR-199a-5p-binding sites. Data are expressed as relative luciferase activity compared to the activity in control samples cotransfected with an equal concentration of CM and correspond to the means ± SEM of three experiments performed in triplicate. *P < 0.05, significantly different from cells cotransfected with CM and the WT or PM 3’UTR.

**FIGURE 7:**
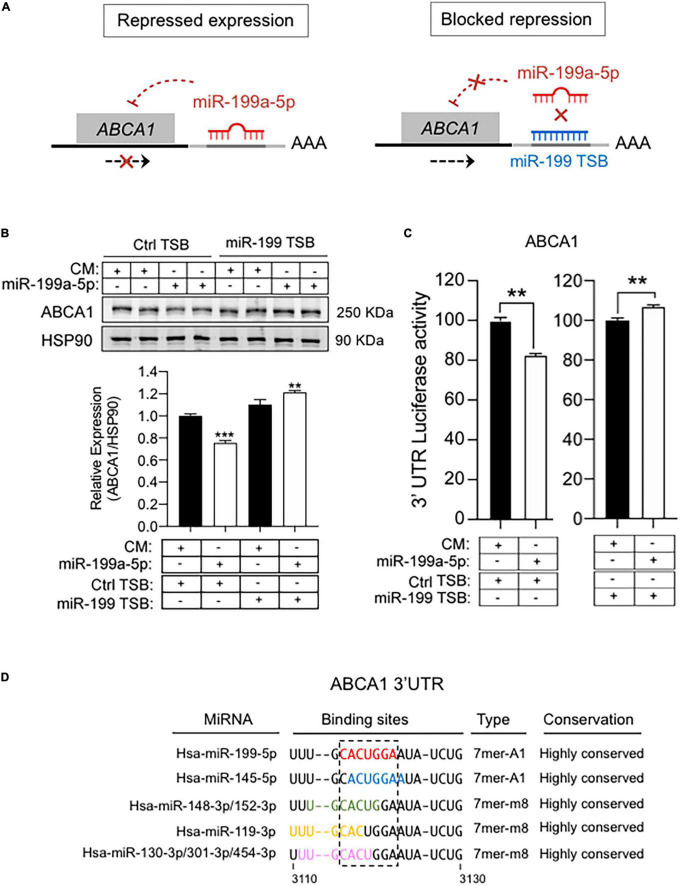
MiR-199 target site blockers and ABCA1 expression. **(A)** Schematic overview of LNA-enhanced target site blocker (TSB) mode of action. Without miR-199 TSB or with scramble TSB, miR-199/RISC complex binds to the 3’UTR of ABCA1 mRNA, thereby attenuating its expression. By contrast, addition of miR-199 TSB blocks the binding of miR-199 and derepress ABCA1. **(B)** Representative Western blot of ABCA1 in mouse peritoneal macrophages transfected as indicated. transfected with synthetic miR-199a-5p mimic in the presence of scramble TSBs or specific miR-199 TSBs for 48 h. Lower panel shows relative ABCA1 protein expression normalized to HSP90 (*n* = 6). *P < 0.05, significantly different from cells cotransfected with CM and Ctrl TSB. **(C)** Luciferase reporter activity in COS-7 cells transfected with CM or miR-199a-5p mimic and the ABCA1 3’UTR in the presence of Ctrl TSB or miR-199a-5p TSB. Data are expressed as relative luciferase and correspond to the means ± SEM of three experiments performed in triplicate. **P < 0.01, significantly different from cells transfected with CM (left panel) or Ctrl TSB (right panel) ***P > 0.001. **(D)** Representation of the human 3’UTR region of ABCA1 where miR-199-5p binding site is located (red), and the overlapping with binding sites for the indicated miRNAs.

To further explore potential regulation of ABCA1 by miR-199a-5p we assessed a bioinformatic analysis that predicted the presence of a putative 7-mer-A1 for miR-199a-5p in the 3′UTRs of ABCA1 (Figure 6A). To confirm the possible impact of this miRNA on the expression of ABCA1, we first transfected mouse peritoneal macrophages with miR-199a-5p mimics or a control non-targeting miRNA (CM) for 48h and ABCA1 expression was stimulated with LXR ligand T0901317 (T090) or Ac-LDL, respectively, for 12 or 24 h before the end of the experiment. As shown in Figure 6B, T090 and Ac-LDL stimulated the expression of ABCA1 in cells transfected with CM, while overexpression of miR-199a-5p significantly blunted ABCA1 induction under the same conditions. More importantly, inhibition of endogenous levels of miR-199a-5p increased ABCA1 expression by T090 or Ac-LDL compared with control conditions (Figure 6C). Given these results, we next sought to analyze the potential mechanism of the posttranscriptional regulation of ABCA1 by miR-199a-5p by assessing the possible direct binding on its predicted site in the 3’UTR which is conserved in human and mouse (Figure 6A). Thus, we cloned the 3’UTR of the ABCA1 gene into a luciferase reporter plasmid and assessed their activity after miR-199a-5p overexpression (Figure 6D). Our data showed that miR-199a-5p significantly repressed ABCA1 3’UTR activity, while the mutation of the miR-199a-5p target site relieved this repression, which is consistent with its direct interaction of this miRNA with the mRNA of ABCA1. To further explore the direct effect of miR-199a-5p on ABCA1 posttranscriptional regulation, we performed loss-of-function experiments using custom-designed miRCURY Target Site Blockers (TSB). As shown in Figure 7A, designed LNA-enhanced antisense oligonucleotides binds to the miR-199-a-5p target site in the 3’UTR region of ABCA1, thereby preventing miRNA from gaining access to that site. Indeed, transfection of miR-199 TSB rescued ABCA1 protein downregulation by miR-199a-5p (Figure 7B) as well as the 3’UTR activity of ABCA1 (Figure 7C). Interestingly, in both experiments we found a slight, but statistical increase (in ABCA1 protein expression and luciferase activity) when combining miR-199a-5p together with miR-199 TSB. This might be mediated by the derepression of several other miRNAs whose binding sites are highly overlapping with miR-199a-5p binding site on the ABCA1 3’UTR region (Figure 7D). Nevertheless, data obtained from these experiments undoubtedly indicate that the inhibition of ABCA1 is due to the direct regulation of ABCA1 3’UTR by miR-199a-5p and rule out indirect effects of miR-199a-5p on other potential regulators of ABCA1 such as Hif1α.

### miR-199a-5p regulates cholesterol efflux to ApoAI

**FIGURE 8:**
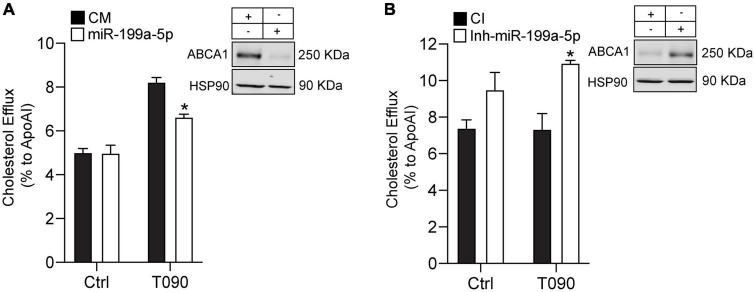
MiR-199a-5p inhibits cholesterol efflux. **(A,B)** Cholesterol efflux analysis to ApoA1 performed in mouse peritoneal macrophages transfected with Ctrl-miR (CM) and miR-199a-5p or Ctrl-Inh (CI) or Inh-miR-199a-5p and stimulated with or without T0901317 (T090) 3 μm for 12 h. Data are the means ± SEM of three independent experiments in triplicate. *P < 0.05 Ctrl-miR-199a-5p compared with miR-199a-5p or Ctrl-Inh-199 compared with Inh-miR-199a-5p. **(A,B)** Representative Western blots of ABCA1 in mouse peritoneal macrophages transfected with CM and miR-199a-5p or CI or Inh-miR-199a-5p. HSP90 was used as a loading control.

The ability of ABCA1 to stimulate the efflux of cholesterol from cells in the periphery, particularly foam cells in atherosclerotic plaques, is an important antiatherosclerotic mechanism (26, 39). Based on our previous findings, we decided to investigate if miR-199a-5p could influence ABCA1 function by assessing cholesterol efflux to ApoA1 during miR-199a-5p overexpression or inhibition in mouse peritoneal macrophages in normoxic conditions. As shown in Figure 8A, miR-199a-5p inhibited cholesterol efflux to ApoA1 and, conversely, inhibition of endogenous levels of this miRNA significantly increased cholesterol efflux through ABCA1 (Figure 8B). Altogether, these results indicate that manipulation of cellular levels of miR-199a-5p alters macrophage cholesterol efflux, a critical step in the reverse cholesterol transport pathway and the development of atherosclerosis.

## Discussion

Numerous evidence supports the pleiotropic effects of hypoxia in the development of different human pathologies from cancer to metabolic diseases (7, 40, 41). In this context, hypoxia is a mechanism that accompanies atherosclerosis progression, which is characterized by an increased accumulation rate of lipids and gives rise to foam cells that populate atherosclerotic plaques. Under normal conditions, macrophages are able to process substantial amounts of lipids and cholesterol without critical overload of the catabolic processes. ABCA1 represents one of the main actors in these processes by mediating cholesterol efflux in macrophages, reverse cholesterol transport (RCT), and HDL biogenesis, as well as inflammatory responses in macrophages (39, 42). As a result, the lack of this transporter or either the inhibition in macrophages has profound implications in the development of atherosclerosis (43). Transcriptional regulation of ABCA1 occurs in a highly regulated manner. When intracellular lipid levels are high, oxidized cholesterols intermediates bind to and activate LXRs, promoting cholesterol efflux to ApoA1 and HDL by regulating ABCA1 and ABCG1, respectively. Similarly HIFα/β also binds to the promoter region of several lipid related genes including ABCA1 in response to hypoxia, which, in turn, activates cholesterol efflux and prevents lipid overload and foam cells, hallmarks of atherosclerosis (14).

However, during progression of atherosclerosis, these pathways become inefficient, leading to imbalance in cholesterol and lipid metabolism and disruption of these cellular functions (42, 44, 45). In the last 10 years, numerous studies have shown that ABCA1 expression is beyond its classic transcriptional regulation, being extensively regulated at the posttranscriptional level by miRNAs including miR-33 (46, 47), miR-758 (26), miR-144 (27), miR-27b (28), miR-148a (29), miR-106b (30), and some RBPs (48). However, there are few evidences of the posttranscriptional inhibition of ABCA1 mediated by hypoxiamiRs. In the present study, we provide data demonstrating that miR-199a-5p and ABCA1 are inversely regulated during hypoxia conditions, and that miR-199a-5p represents a novel posttranscriptional regulator of ABCA1 by in primary mouse macrophages and cholesterol-laden macrophages which pinpoint a possible role in atherosclerosis.

**FIGURE 9:**
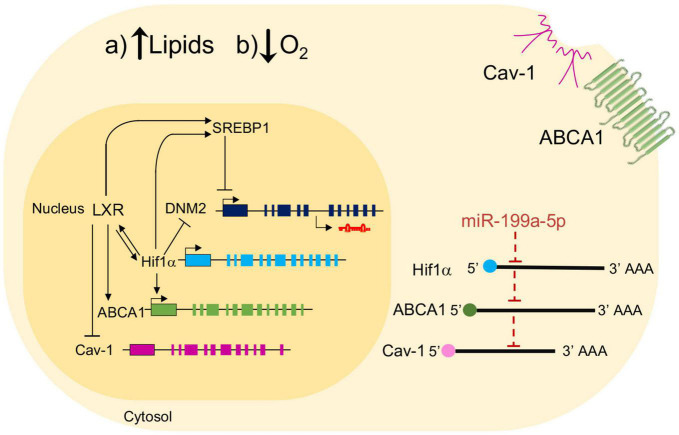
Schematic showing the effect of miR-199a-5p on ABCA1 and potential interplay mechanisms with key genes involved in atherosclerosis. Schematic overview about a possible regulation of miR-199a-5p in the relation between ABCA1, Cav-1, LXR in hypoxia conditions in mouse peritoneal macrophages. Black arrows represent activation processes and inhibition processes. LXR activates several genes involve in atherosclerosis such as ABCA1, SREBP1 and HIF1α, but inhibits Cav-1. On the other hand, some of these genes are inhibited by miR-199a-5p and present a possible direct binding for this miRNA on its predicted site in the 3’UTR.

It has been previously shown that miRNAs regulate ABCA1 during different hypoxic contexts, such as during the pathogenesis of pulmonary arterial hypertension (PAH), where miR-143/145 mediates its posttranscriptional targeting in pulmonary artery smooth muscle cells (PASMCs) contributing to the disease (33). Moreover, in this pathology, miR-20a-5p and its target ABCA1 levels were shown to be inversely correlated (32). Multiple examples in the literature have point out the role of miRNAs in hypoxic situations in macrophages. miR-210, for instance, is upregulated by hypoxia, whereas miR-383 and miR-19a are downregulated (49, 50). Although in the case of miR-199a-5p, a reciprocal regulation between hypoxia and this miRNA has been found in cancer cells (51), to our knowledge, this is the first report showing the relationship between miR-199a-5p and hypoxia in macrophages and ABCA1 regulation. In this line, our experiments in primary mouse macrophages indicate that miR-199a-5p expression is inhibited by hypoxia, which is consistent with previous studies in cardiac myocytes, cirrhotic tissues and the plasma of patients with acute myocardial infarction that showed a decrease of miR-199a-5p expression (36, 37, 52). Indeed, some other previous studies have reported that the inhibition of miR-199a-5p exerts protective effect in the cardiovascular system, specifically in hypoxic cardiomyocytes and myocardium in a ischemia-reperfusion model (53–55). Conversely to miR-199a-5p decrease during hypoxia, we showed that hypoxia upregulated ABCA1 mRNA and protein levels in peritoneal macrophages, as well as other genes implicated in atherosclerosis, including ABCG1 or Cav-1 (34) and other potential predicted targets related to this pathology. In agreement with this, it has been previously demonstrated that ABCA1 is the strongest upregulated gene under these conditions (14). Although miR-199 is a family composed of three members and all could potentially target the same set of genes (35), our analysis evidenced that miR-199a-5p shows a more consistent downregulation than miR-199b-5p. Moreover, increasing exposure to hypoxic conditions was accompanied by gradual miR-199a-5p inhibition while, inversely, levels of ABCA1 protein showed a significant upregulation over time compared to normoxia. In addition to hypoxia, the accumulation of foam cells is critical to the progression of atherosclerosis during the initial stages. In this context, we found that treatment of mouse peritoneal macrophages to promote cholesterol loading using Ac-LDL also inhibited miR-199a-5p expression, while, as expected, ABCA1 levels were raised under this condition. Thus, all these findings evidence an inverse regulation between miR-199a-5p and cholesterol transporters under pro-atherogenic mimicking conditions, to wit, hypoxia and cholesterol accumulation. These results correlated with our analysis showing that overexpression of miR-199a-5p in peritoneal macrophages reduces ABCA1 protein, but more importantly, that the antagonism of miR-199a-5p increases ABCA1 expression. Furthermore, direct binding of miR-199a-5p to the 3’UTR of ABCA1 is consistent to its regulation of ABCA1 by miR-199a-5p at the posttranscriptional levels. On the other hand, similarly to what occurs to ABCA1, hypoxia also induced Cav-1, which is also a direct target of miR-199a-5p (35). In this line, we have previously reported that miR-199a-5p plays an important role in intracellular trafficking as well as cholesterol homeostasis by controlling the expression of key mediators of endocytosis and cholesterol transport including Clathrin (CLC), Cav-1 and the Low Density Lipoprotein Receptor (LDLR) (34). In this regard, correct intracellular membrane trafficking is crucial to ABCA1 expression at the cell surface and facilitates cholesterol efflux in macrophages (56). Based on these findings, it is possible to speculate that the potential contribution of miR-199a-5p in modulating ABCA1 function could be also due to the secondary regulation of additional accessory proteins or the transporter subcellular localization or transcription factors, such as Hif1α, that are simultaneously targets of miR-199 and activators of ABCA1 expression. We ruled out this possibility by using specific target site blocker of the miR-199 binding site on the 3’UTR. Both protein and 3’UTR luciferase activity were rescued when we prevented the binding of miR-199a-5p to the predicted 7mer-A1 site in the ABCA1 3’UTR region. Furthermore, our experiments unquestionably demonstrated that cholesterol efflux to ApoA1 is significantly blunted by miR-199a-5p, whereas inhibition of endogenous levels of this miRNA increases it. Additionally, miR-199a-5p is highly expressed in aorta and cardiometabolic tissues and organs such as aorta, heart, lung in humans, where hypoxia is a critical triggering factor of many associated diseases, including atherosclerosis. In this sense, besides the targeting key cholesterol genes ABCA1 or ABCG1 shown here, some of our recent studies demonstrated that Cav-1 may influence ABCA1 functions (57). Cav-1 is traditionally considered a cholesterol-binding protein that is able to shuttle cholesterol between various cell membranes (58), and acts as a central regulator of cholesterol metabolism during atherosclerosis, which varies depending on the cell type involved. For instance, Cav-1 absence in aortic endothelial inhibits atherosclerosis by attenuating LDL transcytosis and enhancing autophagic flux (59, 60), while in macrophages Cav-1 promotes THP-1 differentiation, ABCA1 expression and cholesterol efflux (57, 61, 62). Indeed, Cav-1 participates in LXR-dependent anti-atherogenic functions including ABCA1 localization, cholesterol efflux as well as the control of inflammatory responses in macrophages (57). Interestingly, it has been reported a crosstalk between LXR and HIF-1α, the primary transcription factor involved in hypoxia and its implication in foam cell formation (63, 64). This is particularly interesting in the context of atherogenesis and reveals a possible correlation between Cav-1, LXR, ABCA1, and hypoxia controlling cholesterol efflux in macrophages, where miR-199a-5p could represent an additional player in this complex scenario (Figure 9). As a possible hint, it has been shown that SREBP1, whose expression is activated by LXR, binds to the promoter region of miR-199a-5p and inhibits its transcription, while SREBP1 contains predicted binding sites for miR-199a-5p (65). In addition to miR-199a-5p, the other strand of miR-199a/b family, the “3p” or passenger strand is reported to be expressed in cardiac tissues adding a layer of complexity to overall regulation. In this sense, a recent article shows that both coexpressed miR-199a-5p and 3p display a diverging target during pathogenic role in cardiac remodeling (66). Even though we have not explored this possibility in hypoxia, an expected role of miR-199a-3p is likely to happen but remains unexplored. Certainly, additional studies will be required to define the potential of the interplay between miR-199a-5p and hypoxia, HIF1 with lipid metabolism and key mediators such as ABCA1, LXR, which could be potentially exploited to therapeutically interfere with the progression of atherosclerosis and cardiovascular associated disease.

## Data availability statement

The raw data supporting the conclusions of this article will be made available by the authors, without undue reservation.

## Ethics statement

This animal study was reviewed and approved by the Yale Animal Care and Use Committee (IACUC).

## Author contributions

AP-G: data curation, formal analysis, and manuscript writing. MT-P, MF-dF, YM-M, VP-M, and PM-G: formal analysis, manuscript writing, and data curation. PM-G: data curation and formal analysis. RB: supervision and manuscript writing. JA: conceptualization, supervision, data curation, and formal analysis. CR: conceptualization, funding acquisition, manuscript writing, data curation, and supervision. All authors contributed to the article and approved the submitted version.

## Footnotes

1
https://genemania.org

2
https://gtexportal.org/

3
http://www.targetscan.org

## Supplementary material

Supplementary Figure 1 ABCA1 expression under combined proaterogenic treatments. Representative Western blot analysis of ABCA1 in mouse peritoneal macrophages under combined treatments with hypoxia ± cholesterol loading with Ac-LDL for 24 h. HSP90 was used as a loading control. Right panel shows relative ABCA1 protein expression normalized to HSP90 (*n* = 3).
