# Supplementary figures and images for: Role of miR-199a-5p in the post-transcriptional regulation of ABCA1 in response to hypoxia in peritoneal macrophages
Source: Front Cardiovasc Med. 2022 Nov 3;9:994080. doi: 10.3389/fcvm.2022.994080 (PMC9669644; doi:10.3389/fcvm.2022.994080)

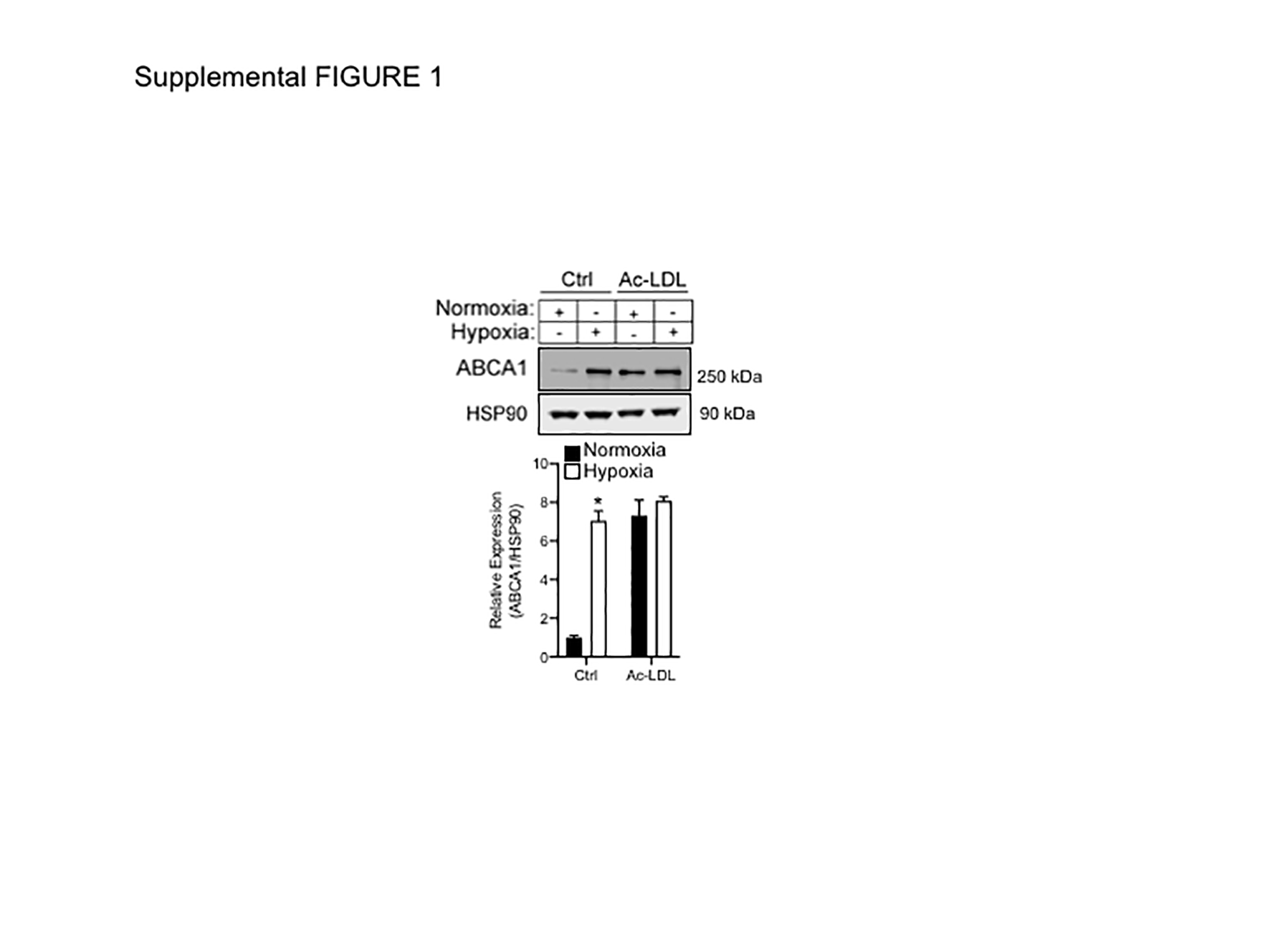

Supplement: Supplementary Figure 1 — ABCA1 expression under combined proaterogenic treatments. Representative Western blot analysis of ABCA1 in mouse peritoneal macrophages under combined treatments with hypoxia ± cholesterol loading with Ac-LDL for 24 h. HSP90 was used as a loading control. Right panel shows relative ABCA1 protein expression normalized to HSP90 (n = 3). [file Image_1.TIFF]
